# Clarification of Safe Delivery by Iranian Experts Based on Clinical Governance: A Qualitative Study

**Published:** 2015-09

**Authors:** Forozun Olfati, Saeid Asefzadeh, Nasrin Changizi, Masud Yonesian, Afsaneh Keramat

**Affiliations:** 1 Student Research Committee, Department of Reproductive Health, Shahroud University of Medical Sciences, Shahroud, Iran; 2 Department of health, Qazvin University of Medical Sciences, Qazvin, Iran; 3 Center for Maternal, Fetal and Neonatal Research, Tehran University of Medical Sciences, Tehran, Iran; 4 Institute for Environmental Research, School of Public Health, Tehran University of Medical Sciences, Tehran, Iran; 5 School of Nursing and Midwifery, Shahroud University of Medical Sciences, Shahroud, Iran

**Keywords:** Clarification, Safe, Delivery, Clinical, Governance, Qualitative research

## Abstract

**Objective:** To clarify the principles of a safe delivery based on Clinical Governance Criteria, as recommended by the pertinent experts.

**Materials and methods:** The current study was part of a qualitative research conducted by content analysis method in 2013 and purposive sampling, performing 24 in-depth interviews based on semi-structured questions and analyzed using thematic content analysis. The participants in this research included midwives, obstetricians, managers, and hospital doctors. The data were under continuous consideration and comparative analysis in order to achieve data saturation.

**Results:** The main concepts derived from interpretations of the pertinent experts include: Patient & Public involvement; Risk Management; Education; Clinical efficiency; Clinical audit; Personnel & Management.

**Conclusion:** In a safe delivery, there is a vicious cycle of causes the elimination of which is only possible through benchmarking patterns that attend to most aspects of a safe delivery. Changes to services require utilization of appropriate change management strategies.

## Introduction

The significance of mothers’ health is because it is one of the eight major goals set for the millennium development (1). Regardless of so many technological achievements of the 21^st^ century, about 44 million pregnant women in developing countries do not have access to prenatal cares services and annually 200000 Asian women die because of the complications of pregnancy and baby delivery. Insufficient prenatal and delivery cares is the cause of mortality for 5 million neonates in each year (2).

Safe delivery is a delivery assisted by educated and skillful individuals in a proper environment which is accessible at an affordable cost and within a short time, where delivery is performed at the highest level of standard and through a proper method, and the result will be a healthy neonate and a healthy mother (3, 4).

Findings of the research performed on “Prenatal Care and pregnancyoutcome” revealed that regardless of the special health care services accessible for pregnant women, the pregnancy outcomes are comparable to the population (5). Although health services are provided for pregnant women in different forms by the therapeutic centers, it seems that there are some factors that inhibit receiving prenatal cares (6, 7). 

According to the research conducted by Moradi Lakeh et al in 2010 in Iran, safe delivery indicators across the country reveal a significant difference between the status quo and the condition of a full compliance with the standards and this is mostly due to social and economic factors (8).

The excessive number of the demands for cesarean operation is a major concern for experts of reproductive health and safe baby delivery and it still remains as an unsolved problem (40%) (2). for every woman who dies, at least 30 suffer injuries and often, permanent disability (9).

The way to address clinical quality has become an important movement all over the world (10).

Clinical governance is a "framework through which health service organizations are accountable for continually improving the quality of their services and safeguarding high standards of care by creating an environment in which excellence in clinical care will flourish" (11). The reason for investing in clinical governance is to improve quality of care and response to public and governmental intolerance of poor performance in health care (12).

In the beginning of 2010, clinical governance office was established in Iran Ministry of Health in order to plan, organize, implement and monitor clinical governance programs also to coordinate clinical governance offices of medical universities all over the country. The seven pillars of clinical governance are as follows: public private involvement, patient safety and risk management, personnel management, education and personnel management, use of information, clinical effectiveness and clinical audit (13, 14). 

Maternity services need to be organized on the principles of ‘availability, accessibility and acceptability’ .The services need to be safe as safety is the back bone of quality. Observance of clinical governance principles helps improving the quality of clinical services in maternity cares (15).

It is believed that there are so many facts that should be dealt with in regard with a safe delivery. To providing a proper ground to improve processes like policy making and planning for health servicesa qualitative research seems necessary in order to find out about the experts’ views in this regard.

## Materials and methods

This is a qualitative content analysis conducted in three cities in Iran including Shahroud, Quazvinand Tehran, in 2013. The content analysis is beyond extraction of visible content taken from textual data. In this study, Colaizzi model was used for content analysis (16). 

Participants in this research include: 10 midwives (Master of Science), 4obstetricians (Specialist physician), 5managers (PhD), 5 hospital doctors (Specialist physician and experts). Totally 28 samples were selected for interview that 4 persons were left out according to lack of interest to continue cooperation, and by interviewers’ discretion. Participants were selected based on their experiences and the research objectives. 

Inclusion Criteria of this study were based on their own tendency to participate in the research and they were all familiar with the concepts of safe baby delivery and clinical governance. Exclusion criteria of this study were based on lack of tendency toward continuing the cooperation, presenting dishonest answers and revoking conscious satisfaction.

Purposive sampling through maximum variation sampling was initially used for data collection. In this sampling method, the basis of selecting participants was having special information about the considered phenomenon, and the aim of their selection was to collect these data. 

On the basis of a goal-oriented sampling managed by the researcher some deep semi-structured interview were held to extract the themes. The duration of each session lasted 1 to 1.5 hours. The interview sessions were repeated several times until the researcher found out that some answers were stated in repetition (data saturation). Sessions were held at the peoples’ workplaces (hospital, maternity center, clinical office, Ministry of Health and Universities).

The questions which were concordant with the goal of the predesigned plan and were used to control the interview sessions and to keep them on clinical governance were as follow:

1. What is a safe delivery, in your opinions?
2. How is the present condition of safe delivery compared with clinical governance standards?
3. What are the inhibitors of a safe delivery?

Interviewer had good communication skills and specialty about interview. Interviewees’ written consent was obtained prior to recording their voices, and the research aims were explained to them. In addition, they were assured that all of their information will remain confidential. All of the participants studied and signed the form of conscious satisfaction designed by the research team. The interviews were conducted in face-to-face approach. 

After each interview, the interview tape was transcribed and analyzed prior to the next interview. Ultimately the findings were compared to the researcher’s interpretations. This finally led to presentation of a deep explanation about the concept of “safe delivery” as believed by the experts. For data analysis, at first, the interview text was studied several times, and then, important sentences were highlighted and codified. Data were considered, and comparative analysis was performed in order to extract primary codes.I n the next stage, themes were organized based on their concept in separate categories. In this part, the primary codes were classified based on differences and similarities in abstract categories and key concept (16). The continuous analysis of data began from the beginning of codification, and continued until the end of data collection. Two of the authors participated in data coding process. The credibility was indebted to the researcher’s sufficient experience, scientific knowledge and academic degree. The MAXQDA_10 _software was employed for management of codes.

All Ethical issues (such as informed consent, conflict of interest, misconduct, co-authorship, double submission, etc.) have been considered carefully. Ethical permission (N0.9227) for the study was obtained from Shahroud University of Medical Sciences on February 17, 2012.

## Results

This study was attended by 24 experts. The findings are presented in the table 1.

The main concepts derived from interpretations of the pertinent experts include: 1.Patient & Public involvement 2. Risk Management 3. Education 4. Clinical efficiency 5. Clinical audit 6. Personnel & Management and 7. Information and its use for a safe delivery.

1. As to the definition of a safe delivery, one of the experts, who was an obstetrician said: “Safe delivery is a cycle beginning with prenatal cares and continues with delivery services, postpartum cares, and services provided for mothers when she is being discharged. On the other hand safe delivery requires the least number of interventions and contributes to the mother’s mental and physical health, esteems her dignity and leaves the least number of complications”. 

2.1. As to the role of public and patient’s participation in safe delivery,a midwife in charge of the ward said “Patient plays the leading role. She should desire and be prepared for pregnancy. She should accept it mentally because this provides her with all influential factors required to improve her condition and ultimately results in accomplishment of the safe delivery”. 

2.2. As to the role played by risk management in a safe delivery, one of the obstetricians uttered “It is unfortunate that it is the files that we manage and not the risks; the tasks are defined at the official surface and are not operational, while risk analysis should be based on accurate recording of the delivery room events”.

2.3. As to the role of education for safe delivery, one of the experts said: “Educational courses held for specialists should be improved. Most midwives still complete continuing education programs but these programs are not of the desirable quality and cannot improve their performance and attitude. Different methods of safe delivery should be practically trained in the wards.” 

2.4. As to the role of Information and its use for a safe delivery, according to the person who was in charge of implementation of clinical governance “Information is collected through daily recording of reports from the wards and from the patients’ files. Some information is sent to the wards in educational folders via automation systems. Service packages and clinical guidelines and instructions are accessible in different work shifts via delivery room’s monitor.”

2.5. As to efficiency of safe delivery, one person who was in charge of clinical governance uttered: The clinical guides are more implemented by nurses and midwives and receive no support from the vice minister of educational affairs. The guide is not emergent and there is no checklist for evaluation. 

2.6. As to clinical audit and the role it has in a safe delivery, one of the experts working at mothers’ health department uttered: Clinical audit is usually a long process during which the main problem remains unsolved. Since only few faults are reported, the clinical audit team cannot deal with the problems efficiently. 

**Table 1:** Themes, major categories, subcategories and codes extracted from the research

**Theme **	**Main Categories**	**Subcategories**	**Codes**
1. Definition of safe delivery	1.1 Educated person (midwife & obstetrician)	1.1. 1. Attending to physical health	- Skillful midwifes have to attend the delivery course. - The delivery should accompany the least amount of risk with the least rate for cesarean.
		1.1.2. mental and emotional health, dignity ,privacy	- There is a need for paying attention to mothers’ mental health dignity and privacy. - Painless delivery methods should be applied. - Midwifes should identify themselves as supporters rather than managers and presenters.
	1.2 environment, facilities and equipment	1.2.1. Equipment	- Monitoring facility must be proportionate to the number of the beds.
		1.2.2. Environment	- The place should be properly designed to be used by patients at any position - The delivery ward should be regarded as the emergency ward.
2. Safe delivery & clinical governance	2.1. The role of public and patient’s involvement	2.1.1. Patient	- Women’s tendency for education can be helpful. - Empowering mothers for making wise decisions and enabling them to identify their needs.
		2.1.2. Society	- Patient’s husband and family, play important roles and how does the society accept a pregnant woman.
	2.2. Role of risk management in a safe delivery	2.2.1. Errors	- Listing risks and communicating them to different wards to prevent them. - Risk reporting method is important. - Staff can anonymously report an error on the site of the hospital information.
		2.2.2. Implementation of risk management	- When high risk pregnancies are identified, the cases have to be followed by high risk mothers system. - A case report system must be established in the ward and personnel must discuss each delivery fault.
	2.3. Role of education in safe delivery	2.3.1. Patient	- Patient’s training can influence the type of delivery she chooses. - Patients have to be trained at the hospitals and at the wards.
		2.3.2. Personnel	- The delivery methods used presently are traditional and students of midwifery and medicine are still learning to apply such methods.
		2.3.3. Specialists	- Seminars, conferences and continuing education programs are required. - Educational courses held for specialists should be improved in quality.
	2.4. Role of information in a safe delivery	2.4.1. Type of Information	- Job skills, personnel’s tasks details, bylaws and guidelines. - Scientific studies must be conducted regarding the issue of safe delivery (analgesic delivery and physiologic delivery).
		2.4.2. Method through which data are obtained	- Some information are sent to the wards through automated system and in educational folders. - Information are given to the personnel through the internet and libraries. - Pregnant women should have access to information through public media and classes
	2.5. Role of efficiency in a safe delivery	2.5.1. Evidence-based medicine	- Due to the large volume of works to be performed by specialists, they do not have time for searching. - Since midwifes are not decision maker, they do not have motivation for searching.
		2.5.2. Clinical guideline	- Clinical guidelines are not operational. - The clinical guideline should be linked to forensic medicine.
	2.6. Role of clinical audit in a safe delivery	2.6.1. Audit cycle	- The system itself is not aware of the contents of the clinical guideline and of the task details. - The approaches recommended by the auditing committees are not operational. - No lessons are learnedfrom the previous accidents and the same faults are repeated. - Sometimes re-auditing is not performed.
		2.6.2. Audit team	- Audit teams are formed only at the nursing level and specialists play an insignificant role in the clinical audit teams.
	2.7. Role of management & personnel in a safe delivery	2.7. 1. Job skill improvement	- The midwife must be experienced in physiological and standard delivery.
		2.7.2. level of job satisfaction	- Payments are not regarded as a sort of investment, rather, they are regarded as cost. - Paying attention to welfare facilities can motivate them for work.
		2.7.3. The number of staff	- Number of personnel is less than the standard.
		2.7.4. Supervisory role of the managers	- Senior managers who make decisions have no clinical experience. - Managers are not sensitive to clinical governance principles.

2.7. As to the role of management & personnel in a safe delivery, a hospital manager said: The obstetricians should be in charge of the measures taken to implement clinical governance. The health system is dependent on one certain individual and all evaluations are carried out by one person while it should be done by the system.

## Discussion

The research findings revealed that there is a vicious cycle of causes and factors that hinders implementation of safe delivery; some of these are: insufficient attention to number of personnel does not meet; empowering mothers for making wise decisions and enabling them to identify their needs; there is no proper culture in the society to propagate the proper type of delivery; paying attention to mothers’ mental health dignity and privacy; Separating low risk of high-risk mothers correctly, lock of sufficient knowledge about clinical governance.

Some of the key facts stated by World Health Organization (WHO) about midwives are as follow: 1- In those parts of the world in which mothers’ mortality rate is low, more than 75% of all deliveries is assisted by the midwives. 2- Complications of delivery and mothers’ mortality rate are reduced for 2.3% in places where educated midwives are employed. 3- In some parts of the world, health care provided by midwives, reduces the need for obstetricians’ intervention for 50%. According to WHO, having access to midwives and equipped medical centers to which patients can be referred, are keys to a safe motherhood and reproductive health (17). 

In a research conducted by Abbaspoor, et al. in 2014 in Iran, although mother’s demand is considered as the major reason for cesarean operation to be so prevalent, actually, mother’s demand is an effect. Socio-cultural, religious and economical norms in the Iranian society play main roles in the selection of the birth method by Iranian women (18).Educated patients tend to have a greater capacity for obtaining, processing, and comprehending basic health information needed to make appropriate health decisions (19).

The privacy right is so significant and is strongly linked to ethical rules and human’s dignity. It is the basic human right which should be enjoyed by all individuals regardless of their position, under any condition, and should be supported by the law. Patients’ privacy has different types: physical privacy, mental privacy and informational privacy (20).

Woogara: “I argue that health professionals can violate patients' privacy in a variety of ways. For example: the right to enjoy their property; the right to protect their medical and personal information as confidential. Some preliminary evidence indicates that many health care practitioners, including nurses, are presently unaware of the articles of the Convention and the implications of the Human Rights Act 1998.In order to prevent litigation for breaches of patients' privacy, it is advocated that universities and other educational institutions should help to produce a clear educational strategy and protocols” (21).

The risk approach to maternity care is supported by the World Health Organization as an efficient tool to reduce mortality during childbirth (22).Risk assessment and risk management on midwifery history has been more considered in the 21st century due to the intensification of risks and disputing nature of the maternal care and legal prosecutions (23).In 1995, clinical risk management standards were defined in England to decrease the costs of delivery failures (24). Risk approach is a method for detecting low-risk pregnancy from high-risk pregnancy. In Iran, the separation of high-risk mothers from low-risk mothers at health centers is performed better than at hospitals.

Graham, in his article in 2009 stated that in low-income countries improving quality of maternity care through criterion-based clinical audit can be effective. According to this method, we can provide a list of standard criteria for clinical audit, and accordingly did (25). Clinical audit is an important part of quality assurance and is based on two major principles: 1- Commitment for better performance, which means continuous improvement of audit findings, 2- acceptation of the concept of the best action or evidence based action, which means that physicians and midwives can manage the conditions in the best way (26). Clinical audit is a fundamental way to conduct proper clinical guidelines.

## Conclusion

In summary: More studies are needed to be conducted on patients’ privacy and esteeming it; a committee should be formed to be attended mainly by obstetricians in order to decrease the number of cesarean operations; Considering the fact that clinical governance has been accepted as the means of improving quality of services, it is proper to define the delivery ward’s protocol on the basis of the axes of clinical governance. 

## References

[1] Sachs JD, McArthur JW (2005). The millennium project: a plan for meeting the millennium development goals. The Lancet.

[2] Organization WHO Country Cooperation Strategy for WHO and Islamic Republic of Iran 2010– 4.

[3] The Royal College of Midwives, The Royal College of Anaesthetists (2007). The Royal College of Paediatrics and Child Health. Safer Childbirth: Minimum standards for the organisation and delivery of care in labour.

[4] AbouZahr C (2003). Safe Motherhood: a brief history of the global movement 1947–2002. British Medical Bulletin.

[5] Quick J, Gteenlick M, Roghmann K (1981). Prenatal Care and Pregnancy Outcome in an HMO General Population: A Multivariate Cohort Analysis. AJPH.

[6] Simkhada B, Teijlingen ER, Porter M, Simkhada P (2008). Factors affecting the utilization of antenatal care in developing countries: systematic review of the literature. JAdv Nurs.

[7] Lotfi R (2004). Quality of prenatal care in LQAS method in Astara health centers method. Guilan University of Medical Sciences Journal.

[8] Moradi-Lakeh M, Ramezani M, Naghavi M (2007). Equality in safe delivery and its determinants in Iran. Archives of Iranian medicine.

[9] Donnay F (2000). Maternal survival in developing countries: what has been done, what can be achieved in thenext decade. International Journal of Gynecology & Obstetrics.

[10] Scally G, Donaldson LJ (1998). The NHS's 50 anniversary.Clinical governance and the drive for quality improvement in the new NHS in England. Bmj.

[11] Malcolm L, Mays N (1999). New Zealand's independent practitioner associations: a working model of clinical governance in primary care?. Bmj.

[12] Wright L, Barnett P, Hendry Ch (2001). Clinical leadership and clinical governance, report commissioned by the clinical.

[13] Ravaghi H, Mohseni M, Rafiei S, Zadeh NS, Mostofian F, Heidarpoor P (2014). Clinical Governance in Iran: Theory to Practice. Procedia - Social and Behavioral Sciences.

[14] Hooshmand E, Tourani S, Ravaghi H, Ebrahimipour H (2014). Challenges in evaluating clinical governance systems in iran: a qualitative study. Iranian Red Crescent medical journal.

[15] Arulkumaran S (2010). Clinical governance and standards in UK maternity care to improve quality and safety. Midwifery.

[16] Streubert HJ, Carpenter DR (2003). Qualitative research in nursing، advancing the humanistic imperative.

[17] World Health Organization (1990). Human resource development for maternal health and safe motherhood : report of a task force meeting.

[18] Abbaspoor Z, Moghaddam -Banaem L, Ahmadi F, Kazemnejad A (2014). Iranian mothers' selection of a birth method in the context of perceived norms: A content analysis study. Midwifery.

[19] Arora NK, McHorney CA ( 2000). Patient preferences for medical decision making: who really wants to participate?. Medical care.

[20] Woogara J (2005). Patients’ rights to privacy and dignity in the NHS. Nurs Stand.

[21] Woogara J (2001). Human rights and patients’ privacy in UK hospitals. Nursing Ethics.

[22] Backett EM, Davies AM, Petros-Barvazian A (1984). The risk approach in health care: with special reference to maternal and child health, including family planning.

[23] Kenyon C (2009). Risk management standards in midwifery are no substitute for personal knowledge and accountability. Complement TherClinPract.

[24] Winn SH (2007). Assessing and credentialing standards of care: the UK Clinical Negligence Scheme for Trusts (CNST, Maternity). Best Practice & Research Clinical Obstetrics &Gynaecology.

[25] Graham WJ (2009). Criterion-based clinical audit in obstetrics: bridging the quality gap?. Best Pract Res ClinObstetGynaecol.

[26] Bullough C, Graham W (2004). Clinical audit learning from systematic case reviews against explicit criteria.

